# Diabetes Is Associated with Musculoskeletal Pain, Osteoarthritis, Osteoporosis, and Rheumatoid Arthritis

**DOI:** 10.1155/2019/6324348

**Published:** 2019-12-06

**Authors:** Thomas Rehling, Anne-Sofie Dam Bjørkman, Marie Borring Andersen, Ola Ekholm, Stig Molsted

**Affiliations:** ^1^Department of Clinical Research, Nordsjællands Hospital, Denmark; ^2^National Institute of Public Health, University of Southern Denmark, Denmark

## Abstract

**Aim:**

To investigate the associations between diabetes and musculoskeletal pain, osteoarthritis, osteoporosis, and rheumatoid arthritis.

**Methods:**

Self-reported data were provided by the nationwide Danish National Health Survey 2013. Inclusion criteria were age ≥ 40 years and known diabetes status. The exposure variable was diabetes, and the outcome variables included musculoskeletal pain during the last 14 days in three body sites (back/lower back, limbs, and shoulder/neck), osteoarthritis, osteoporosis, and rheumatoid arthritis. Logistic regression analyses adjusted for age, gender, BMI, education, marital status, and physical activity were performed.

**Results:**

9,238 participants with diabetes were 65.6 ± 11.0 (mean ± SD) years old; 55.6% were males. 99,980 participants without diabetes were 59.2 ± 11.8 years old; 46.7% were males. Diabetes was associated with back/lower back pain (OR 1.2 (CI 95% 1.1-1.2), *p* < 0.001), pain in the limbs (1.4 (1.3-1.4), *p* < 0.001), shoulder/neck pain (1.2 (1.1-1.3), *p* < 0.001), osteoarthritis (1.3 (1.2-1.4), *p* < 0.001), osteoporosis (1.2 (1.1-1.4), *p* = 0.010), and rheumatoid arthritis (1.6 (1.4-1.7), *p* < 0.001). In participants with diabetes, physical activity was associated with reduced pain (e.g., back/lower back pain (0.7 (0.6-0.7), *p* < 0.001)).

**Conclusion:**

Diabetes was associated with elevated odds of having musculoskeletal pain. Diabetes was also associated with elevated odds of having osteoarthritis, osteoporosis, and rheumatoid arthritis. The most frequent disease in individuals with diabetes was osteoarthritis. The reported pain may have negative impacts on the level of physical activity. Health-care professionals should remember to inform patients with diabetes that musculoskeletal pain, osteoarthritis, osteoporosis, and rheumatoid arthritis are not contraindications to exercise training.

## 1. Introduction

Diabetes is associated with medical complications and comorbidities that increase the relative risks of mortality and morbidity [1]. The diabetes-related complications and comorbidities are not only expensive to treat but also have negative impacts on the patients' quality of life [2].

Pain from the musculoskeletal system is a frequent problem in patients with diabetes as it is reported more often by individuals with diabetes than in the general population [3]. In a previous study on 950 patients with type 2 diabetes (type 2 DM), musculoskeletal pain ranged from 1.7 to 2.1 times as frequent as in an age- and gender-matched general population [3]. The study found that pain was associated with higher BMI, reduced quality of life, low physical function, and the ability to be physically active. Other studies have also reported that diabetes is associated with an increased risk of having musculoskeletal pain [4, 5].

Musculoskeletal pain in individuals with diabetes may arise from several comprising factors, among them osteoarthritis. Previous studies have reported the associations between diabetes and osteoarthritis [6, 7]. The overweight and obesity that are often observed in patients with diabetes may be an important factor in the development of osteoarthritis, especially in the development of osteoarthritis in the back and the lower limbs where an elevated bodyweight will increase the load on the joints. The pain may also be a result of osteoporosis which is associated with type 2 DM via a vitamin D deficiency [8]. Furthermore, musculoskeletal pain in individuals with diabetes is also found in joints and their surrounding tissues as the results of advanced glycation end-stage (AGE) products [9]. The pain may also arise from the frequent complication of diabetic polyneuropathy.

Physical activity is a recognized part of the treatment of type 2 DM. The effects of exercise training in individuals with type 2 DM may include an improved glycaemic control, reduced blood pressure, improved dyslipidaemia, and a reduced BMI [10–12]. Thus, exercise training has the potential to decrease the risks of diabetic complications and mortality in patients with type 2 DM. However, the level of physical activity in individuals with type 2 DM remains reduced compared to that in individuals without type 2 DM [13]. Individuals with diabetes may have several barriers to being physically active, among them musculoskeletal pain [14]. Thus, if musculoskeletal pain affects physical activity levels negatively in patients with diabetes, it may have more negative implications including impaired glycaemic control and a reduced physical function.

Whilst musculoskeletal pain in individuals with diabetes has been reported in previous studies, the reasons underlying the pain are less clear. This study hypothesized that diabetes was associated with elevated odds of having musculoskeletal pain, osteoarthritis, osteoporosis, and rheumatoid arthritis. The aim of this study was to investigate the association between diabetes and musculoskeletal pain and the associations between diabetes and osteoarthritis, osteoporosis, and rheumatoid arthritis.

## 2. Subject Material and Methods

Data were provided by the nationwide Danish National Health Survey 2013 (with the same methodology as in [15]). The survey assesses trends in health and morbidity in the adult Danish population and investigates factors that are associated with health status and health behaviour. The participants in the survey were randomly invited from the general population using the Danish Civil Health Registration System. All citizens in Denmark receive a number from the Danish Civil Health Registration System, and a total registration of citizens in the country is thereby possible. Out of 300,450 individuals, 162,283 (54%) completed the self-administered questionnaire. To assure that the participants represent the national population, a calibration weighting was used in the analyses (see Statistical Analyses). All data were self-reported. The study was approved by the Danish Data Protection Agency.

In the present study, participants from the original study (the Danish National Health Survey 2013) were included if they were ≥40 years of age. Diabetes types were not reported as a nature of the original survey. Participants below 40 years of age were excluded to minimize the number of participants with type 1 diabetes mellitus in the cohort, as the majority of individuals with diabetes in the younger population has type 1 diabetes mellitus [16]. Thus, the majority of the participants with diabetes were anticipated to have type 2 DM.

Three questions were used to assess pain from the musculoskeletal system during the past 14 days: *Pain in the shoulder and neck*, *Pain in the back and lower back*, and *Pain in the arm, hand, knee and/or hip* (the limbs) [15]. The three questions had the answer categories: *Yes, very bothered*; *Yes, bothered a little*; or *No*. The reported answers of pain were in each of the three questions recoded into the dichotomous answers “yes” (*Yes, very bothered*; *Yes, bothered a little*) and “no” according to the procedure used by the Danish National Health Survey.

The participants' highest level of education was used as a proxy for socioeconomic position: low (<10 years of education), middle (10-12 years of education), high (≥13 years of education), or other (students or individuals with an uncategorized foreign education).

Leisure time physical activity level was assessed using the four level Saltin-Grimby Physical Activity Level Scale [17]. The participants were asked the question “*If we look at the past year, what would you say best describes your spare-time activities?*”. The question had four response categories: (1) *high intensity sports several times during a week*, (2) *minimum four hours of exercise training weekly*, (3) *minimum four hours of moderate activity as walking or cycling weekly*, and (4) *read, watch television or other sedentary activities*. The participants were stratified into physically active and physically inactive individuals by pooling the three aforementioned highest levels of physical activity into one level (from moderate to high intensity) versus the last group with sedentary activities, respectively. The participants' age, gender, BMI, marital status, and ethnic background were also included.

### 2.1. Statistical Analyses

The associations between the exposure variable diabetes and the outcome variables pain, osteoarthritis, osteoporosis, and rheumatoid arthritis were tested in multiple logistic regression analyses. In the analyses of the associations between diabetes and pain, osteoarthritis, osteoporosis, and rheumatoid arthritis, the models were adjusted for age, gender, BMI, education, marital status, and physical activity to control for confounding. In the analyses of the association between physical activity and pain, osteoarthritis, osteoporosis, and rheumatoid arthritis in participants with diabetes, the models were adjusted for age, gender, BMI, education, and marital status to control for confounding. Unadjusted and adjusted models are presented in Results. Data for the regression analyses are presented as the odds ratio (OR) and 95% confidence intervals (CI). Descriptive demographic and clinical data are presented as numbers and percentages or the mean ± standard deviation (SD). *p* < 0.05 was statistically significant.

Calibration weighting was included to reduce the potential impact of nonresponse bias on the estimates. The weights were computed by Statistics Denmark based on the collected information of variables, among them gender, age, marital status, level of education, income, employment status, country of origin, health-care utilisation, and research protection for all individuals who were invited [18].

## 3. Results

Out of the 162,283 respondents, 109,218 were 40 years of age or older and reported diabetes status. Diabetes was reported by 9,238 (8.5%) participants, and 99,980 (91.5%) reported that they had no diabetes. Characteristics of the participants are presented in Table 1. The overall mean age was 59.7 ± 11.9 years, 51.8% were men, and 85.4% were married or living with a partner. Age was associated with all musculoskeletal pain variables (e.g., back/lower back pain OR 1.0 (1.0-1.0), *p* < 0.001), and osteoarthritis, osteoporosis, and rheumatoid arthritis (e.g., osteoarthritis OR 1.1 (1.1-1.1), *p* < 0.001). Gender, BMI, and marital status were also associated (*p* < 0.001) with all musculoskeletal pain variables, osteoarthritis, osteoporosis, and rheumatoid arthritis (data not shown).

The frequency of musculoskeletal pain, osteoarthritis, osteoporosis, and rheumatoid arthritis are presented in Table 2. The pain in the limbs was found to be the most frequently reported pain in participants with or without diabetes. Osteoarthritis was more frequently reported than osteoporosis and rheumatoid arthritis.

Table 3 shows that diabetes was significantly associated with back/lower back pain (adjusted OR 1.2 (1.1-1.2), *p* < 0.001), pain in the limbs (adjusted OR 1.4 (1.3-1.4), *p* < 0.001), and shoulder/neck pain (adjusted OR 1.2 (1.1-1.3), *p* < 0.001). Furthermore, diabetes was also found to be associated with osteoarthritis (adjusted OR 1.3 (1.2-1.4), *p* < 0.001), osteoporosis (adjusted OR 1.2 (1.1-1.4), *p* = 0.010), and rheumatoid arthritis (adjusted OR 1.6 (1.4-1.7), *p* < 0.001) (Table 4). In participants with diabetes, physical activity was associated with reduced back/lower back pain (adjusted OR 0.7 (0.6-0.7), *p* < 0.001), pain in the limbs (adjusted OR 0.6 (0.5-0.7), *p* < 0.001), and shoulder/neck pain (adjusted OR 0.8 (0.7-0.8) *p* < 0.001) (Table 5).

## 4. Discussion

The findings in this study were that diabetes was associated with elevated odds of having musculoskeletal pain and the diseases osteoarthritis, osteoporosis, and rheumatoid arthritis. Osteoarthritis was more frequently reported than osteoporosis and rheumatoid arthritis in participants with or without diabetes. In participants with diabetes, being physically active was associated with reduced odds of having musculoskeletal pain.

Diabetes was reported by 8.5%, a prevalence that exceeded the overall Danish diabetes prevalence of ~5.1% [19]. The elevated prevalence of diabetes in this study was a result of the inclusion of individuals above 40 years of age only, where the type 2 DM prevalence rises. Diabetes was associated with elevated odds of having musculoskeletal pain, and this finding serves to underscore the importance of having a focus on pain in clinical practice. Attention given to musculoskeletal pain in diabetes treatment should especially be remembered when an intervention aims to increase the level of physical activity. Whilst musculoskeletal pain may reduce the motivation to increase physical activity levels if it hurts during activity, it may also have a negative impact on the adherence to exercise training programs. Musculoskeletal pain may also affect the quality of life negatively. Thus, in the diabetes treatment, it is relevant to assess musculoskeletal pain to combat barriers to physical activity and general well-being.

The most pronounced association between diabetes and pain was found in the limbs, which also was the most frequently reported pain in participants with diabetes. The result is supported by the data from a previous study on participants with type 2 DM where the same pain assessment method was used [3]. The pain in the limbs may be symptoms of osteoarthritis in the knees and hips, body sites where osteoarthritis often is registered [20]. However, the pain in the limbs may also arise from the diabetes-specific complication of peripheral neuropathy or manifestations of the diabetes-specific pathogenesis in AGE. Indeed, musculoskeletal manifestations that arise as the results of AGE may also occur together with osteoarthritis and thereby have additive destructive effects on joints and their surrounding tissues.

As anticipated, osteoarthritis was the most frequently reported disease in the participants with and without diabetes. To these authors' knowledge, this study used the largest population sample to investigate the association between diabetes and osteoarthritis. Diabetes was associated with 28% increased odds of having osteoarthritis, a finding that is similar to results from other studies [6, 7]. The association was found even after adjustments for BMI and level of physical activity. When osteoarthritis occurs with diabetes, it may have additive negative impacts on financial costs and patient disability [21]. As this was a cross-sectional study with limited types of data, it was not possible to investigate the causal mechanism between diabetes and osteoarthritis. A recent study suggested a plausible explanation. Individuals without diabetes aged 55 years were included in a cohort study and tested again after 16-18 years [22]. The study found that osteoarthritis in the knees or hips were independent predictors of incident diabetes. The development of diabetes was partly explained by physical inactivity due to walking limitations as a result of osteoarthritis. Whether type 2 DM has the potential to develop osteoarthritis is unknown. However, low-grade inflammation is a well-known issue in diabetes [23] and osteoarthritis, and it may link the two diseases [24]. The inflammation may arise from metabolic factors, among them visceral obesity and dyslipidaemia, and it leads to increased risks of type 2 DM and osteoarthritis [25, 26].

The most pronounced association between diabetes and the other reported diseases was found in the association between diabetes and rheumatoid arthritis, and diabetes was associated with 55% elevated odds of having rheumatoid arthritis. However, in this study, rheumatoid arthritis was reported by 15.1% and 7.6% of the participants with and without diabetes, respectively. The high prevalence of rheumatoid arthritis in this study is most likely based on a misunderstanding of the disease among the participants. More participants may not know the difference between osteoarthritis and rheumatoid arthritis, which have comparable names in Danish, as the prevalence in the two groups exceeds previously reported numbers of rheumatoid arthritis [27]. Thus, the present data for rheumatoid arthritis should be interpreted with great caution. A previous study found that type 2 DM was associated with an increased risk of having rheumatoid arthritis in women [28]. If rheumatoid arthritis appears before diabetes, the pain from rheumatoid arthritis may increase the risk of physical inactivity, which is a type 2 DM risk factor. In addition to that, it should not be ignored that long-term steroid treatment of rheumatoid arthritis could increase the risk of type 2 DM, a mechanism that may be accelerated with physical inactivity.

The analysis of the association between physical activity and musculoskeletal pain suggested that there may be a positive effect of physical activity on reduced musculoskeletal pain, an effect that has been documented in patients with osteoarthritis in the knee [29] and the hip [30]. It is, however, unknown whether exercise training decreases musculoskeletal pain in patients with type 2 DM. Whilst pain may affect mental health negatively [31], the musculoskeletal pain in diabetes may also be enhanced by impaired mental health. Patients with type 2 DM have an elevated risk of depression and anxiety, and these mental issues may worsen the pain sensation [32]. However, exercise training has the potential to decrease chronic inflammation [33]. Thus, exercise training may not only prevent type 2 DM but also be part of the treatments of type 2 DM and osteoarthritis.

The present study was limited by the following reasons: all data were self-reported, and especially the reported diseases may be biased. Other limitations were that the diabetes types were not reported and more clinical variables would also have been interesting to include in the data analyses. Furthermore, the data were collected in a national study using a cross-sectional design, which does not allow conclusions with regard to causality. A strength of the study was the relatively great number of participants from a nationwide survey.

This study has important clinical implications. First, individuals with diabetes have an elevated risk of having musculoskeletal pain, a problem that needs attention in clinical practice especially in relation to interventions that aim to increase physical activity levels. Second, individuals with diabetes have elevated risks of having osteoarthritis where exercise training is not contraindicated but recommended to decrease symptoms, among them pain. Thus, individuals with diabetes and osteoarthritis may not only receive positive effects of physical activity or exercise training on glycaemic control, as physical activity and exercise training may also reduce pain from the musculoskeletal system. Health-care professionals including medical doctors, nurses, physiotherapists, and exercise practitioners with contacts to individuals with diabetes should remember informing the patients about the importance of a physically active lifestyle, even though in diabetes, one of the three reported diseases occurs in the patient.

## 5. Conclusion

In conclusion, diabetes was associated with musculoskeletal pain, osteoarthritis, osteoporosis, and rheumatoid arthritis. Osteoarthritis was more frequently reported than osteoporosis and rheumatoid arthritis. This study suggests having a focus on musculoskeletal pain in individuals with diabetes in clinical practice and informing those with diabetes and osteoarthritis, osteoporosis, or rheumatoid arthritis that exercise training may not only have positive impacts on pain from the musculoskeletal system but also on glycaemic control.

## Figures and Tables

**Table 1:** Characteristics of participants with diabetes or without diabetes.

	Diabetes (*n* = 9,238)	No diabetes (*n* = 99,980)
Age (years)	65.6 ± 11.0	59.2 ± 11.8
Sex		
Men	5,137 (55.6)	46,658 (46.7)
Women	4,101 (44.4)	53,322 (53.3)
Marital status		
Married/living with partner	6,544 (70.8)	78,899 (78.9)
Living alone	2,694 (29.2)	21,081 (21.1)
Socioeconomic position		
Low	1,818 (22.4)	10,845 (11.6)
Middle	3,951 (48.8)	47,375 (50.6)
High	1,735 (21.4)	30,890 (33.0)
Other	597 (7.4)	4,488 (4.8)
BMI (kg/m^2^)	28.9 ± 5.5	25.8 ± 4.4

Data are presented as number (%) or mean ± SD.

**Table 2:** Musculoskeletal pain, osteoarthritis, rheumatoid arthritis, osteoporosis, and physical activity in participants with diabetes or without diabetes.

	Diabetes (*n* = 9,238)	No diabetes (*n* = 99,980)
Musculoskeletal pain		
Back/lower back pain	5,411 (60.6)	50,548 (51.4)
Pain in the limbs	6,628 (73.8)	59,822 (60.7)
Shoulder/neck pain	4,978 (56.0)	50,752 (51.5)
Rheumatic disease/arthritis		
Osteoarthritis	3,679 (43.5)	28,910 (29.4)
Osteoporosis	528 (6.4)	4,765 (4.8)
Rheumatoid arthritis	1,219 (15.1)	7,378 (7.6)
Physically active	6,220 (71.6)	83,431 (85.8)

Data are presented as number (%).

**Table 3:** Multiple logistic regression on the associations between diabetes and musculoskeletal pain.

	Back/lower back pain		Pain in the limbs		Shoulder/neck pain	
Unadjusted model						
Exposure	OR (95% CI)	*p*	OR (95% CI)	*p*	OR (95% CI)	*p*
Diabetes	1.5 (1.4-1.6)	<0.001	2.0 (1.9-2.1)	<0.001	1.3 (1.2-1.3)	<0.001
Adjusted model^∗^						
Exposure	OR (95% CI)	*p*	OR (95% CI)	*p*	OR (95% CI)	*p*
Diabetes	1.2 (1.1-1.2)	<0.001	1.4 (1.3-1.4)	<0.001	1.2 (1.1-1.3)	<0.001

^∗^Adjusted for age, gender, BMI, education, marital status, and physical activity.

**Table 4:** Multiple logistic regression on the associations between diabetes and osteoarthritis, rheumatoid arthritis, and osteoporosis.

	Osteoarthritis		Osteoporosis		Rheumatoid arthritis	
Unadjusted model						
Exposure	OR (95% CI)	*p*	OR (95% CI)	*p*	OR (95% CI)	*p*
Diabetes	2.0 (1.9-2.1)	<0.001	1.5 (1.3-1.6)	<0.001	2.3 (2.1-2.5)	<0.001
Adjusted model^∗^						
Exposure	OR (95% CI)	*p*	OR (95% CI)	*p*	OR (95% CI)	*p*
Diabetes	1.3 (1.2-1.4)	<0.001	1.2 (1.1-1.4)	0.010	1.6 (1.4-1.7)	<0.001

^∗^Adjusted for age, gender, BMI, education, marital status, and physical activity.

**Table 5:** Multiple logistic regression on the associations between physical activity and musculoskeletal pain in participants with diabetes (*n* = 9,238).

	Back/lower back pain		Pain in the limbs		Shoulder/neck pain	
Unadjusted model						
Exposure	OR (95% CI)	*p*	OR (95% CI)	*p*	OR (95% CI)	*p*
Physical activity	0.6 (0.5-0.7)	<0.001	0.5 (0.5-0.6)	<0.001	0.8 (0.7-0.9)	<0.001
Adjusted model^∗^						
Exposure	OR (95% CI)	*p*	OR (95% CI)	*p*	OR (95% CI)	*p*
Physical activity	0.7 (0.6-0.7)	<0.001	0.6 (0.5-0.7)	<0.001	0.8 (0.7-0.9)	<0.001

^∗^Adjusted for age, gender, BMI, level of education, and marital status.

## Data Availability

The data were provided by the Health and Morbidity Survey after a request from the author SM. The majority of the data presented in this study are presented for groups of participants by the Health and Morbidity Survey (http://www.danskernessundhed.dk/). Data for the individual participants cannot be accessed as they are protected by the Data Protection Agency.
